# Formulation and *In Vitro* Evaluation of Casein Nanoparticles as Carrier for Celecoxib

**DOI:** 10.34172/apb.2020.049

**Published:** 2020-05-11

**Authors:** Jyotsana R. Madan, Izharahemad N. Ansari, Kamal Dua, Rajendra Awasthi

**Affiliations:** ^1^Department of Pharmaceutics, Smt. Kashibai Navale College of Pharmacy, Savitribai Phule Pune University, Pune 411048, Maharashtra, India.; ^2^Discipline of Pharmacy, Graduate School of Health, University of Technology Sydney, Ultimo NSW 2007, Australia.; ^3^Amity Institute of Pharmacy, Amity University Uttar Pradesh, Noida 201313, India.

**Keywords:** Casein nanoparticles, Celecoxib, Nanocarrier, Reassembled casein micelles, Sodium caseinate

## Abstract

***Purpose:*** The objective of this work was to formulate casein (CAS) nanocarriers for the dissolution enhancement of poorly water soluble drug celecoxib (CLXB).

***Methods:*** The CLXB loaded CAS nanocarriers *viz*., nanoparticles, reassembled CAS micelles and nanocapsules were prepared using sodium caseinate (SOD-CAS) as a carrier to enhance the solubility of CLXB. The prepared formulations were characterized for particle size, polydispersity index, zeta potential, percentage entrapment efficiency, and surface morphology for the selection of best formulation. Fourier transform infrared spectroscopy, differential scanning calorimetry and X-ray powder diffraction study was used to for the confirmation of encapsulation of CLXB. Further,*in vitro* drug dissolution, *ex-vivo* permeation studies on chicken ileum and stability studies were carried out.

***Results:*** The CLXB loaded casein nanoparticles (CNP) (batch A2) showed a particle size diameter 216.1 nm, polydispersity index 0.422 with percentage entrapment efficiency of 90.71% and zeta potential of -24.6 mV. Scanning electron microscopy of suspension confirmed globular shape of CNP. The*in vitro* release data of optimized batch followed non Fickian diffusion mechanism. The *ex vivo* permeation studies on chicken ileum of CLXB loaded CNP showed permeation through mucous membrane as compared to pure CLXB. The apparent permeability of best selected freeze dried CLXB loaded CNP (batch A2) was higher and gradually increased from 0.90 mg/cm^2^ after 10 min to a maximum of 1.95 mg/cm^2^ over the subsequent 90 min. A higher permeation was recorded at each time point than that of the pure CLXB.

***Conclusion:*** The study explored the potential of CAS as a carrier for solubility enhancement of poorly water soluble drugs.

## Introduction

Casein (CAS) is an inexpensive, readily available, non-toxic and highly stable milk protein. This protein is biocompatible, biodegradable and amphiphilic in nature and generally recognized as safe as natural food.^1^ The structural and physicochemical properties of CAS facilitate its functionality in drug delivery systems. These properties include binding of ions and small molecules, exceptional surface-active and stabilizing properties, excellent emulsification and self-assembly properties together with superb gelation and water binding capacities. CAS proteins have well defined hydrophobic and hydrophilic areas, which can self-assemble in the form of stable micelles in aqueous solutions.^2-6^ The strong association property of CAS is favorable for the nanoencapsulation processes. CAS-based nanoparticles are being recognized as potential delivery vehicles for nutraceuticals and pharmaceutical materials.^7-11^

Celecoxib (CLXB) is a non-steroidal anti-inflammatory drug that was recognized as a specific inhibitor of COX-2 and approved by the United States Food and Drug Administration for the treatment of different types of joint inflammation and intense pain. In addition, it was recently approved as an oral adjunct to prevent colon cancer development in patients with familial adenomatous polyposis. It has been also investigated for its chemotherapeutic potential in the therapy of advanced cancers.^12-14^ According to Biopharmaceutical Classification System; CLXB is classified as a low solubility and high permeability drug (class II). The particle size of CLXB influences the content uniformity, dissolution and bioavailability of the product. The t_max_ of CLXB is about three hours after oral administration. Rapid onset of action is necessary to provide fast pain relief in the treatment of acute pain. Therefore, it is necessary to enhance the aqueous solubility and dissolution rate of CLXB to obtain faster onset of action, to minimize the variability in absorption and improve its overall oral bioavailability. This can be achieved by developing a nano-formulation of the CLXB.^15,16^

In this work, we propose an oral drug delivery system for CLXB based on the use of CAS, which has been well recognized as a promising biomaterial to develop nanocarriers for oral drug delivery. For this, nanoparticle, reassembled CAS micelles and CAS nanocapsules were synthesized to load CLXB to obtain a sustained drug release profile of CLXB. The prepared nanoformulations were characterized for particle size, polydispersity index, zeta potential, percentage entrapment efficiency, and surface morphology and *in vitro* drug dissolution. *Ex-vivo* permeation study of was carried out on chicken ileum. Finally, the best selected formulation was evaluated for storage stability to understand the effect of storage conditions.

## Materials and Methods

### Materials

CLXB was gifted by Aarti Drugs Ltd., Tarapur, India. Sodium caseinate (SOD-CAS) was gifted by Clarion Casein Ltd., North-Gujarat, India. Dialysis Membrane-110 was purchased from Himedia Laboratories Pvt. Ltd., Mumbai, India. Potassium phosphate, tri-potassium citrate, calcium chloride, sodium hydroxide, acetone, soya lecithin and sodium lauryl sulfate were purchased from Research-Lab Fine Chem Industries, Mumbai, India. All other reagents were of analytical grade and used as received.

### Methods

#### Preparation of simulated intestinal fluid (SIF, USP, without pancreatin) with sodium lauryl sulphate (SIFSLS 1%)

Potassium dihydrogen phosphate (6.805 g), sodium hydroxide (0.896 g) and sodium lauryl sulfate (10 g) were dissolving in sufficient amount of deionized water to produce 1000 mL of 1% SIFSLS. This solution was further used as a release medium.^17^

### Solubility studies

Solubility was determined by placing ≈50 mg of CLXB in 5 mL each of distilled water, 1% SIFSLS, phosphate buffers (pH 6.8). Solutions were stirred using a magnetic stirrer (REMI Motors, Mumbai, India) at 200 rpm for 24 h. The temperature was maintained at 25 °C. The solutions were filtered through Whatman filter paper No. 41 and the CLXB concentration was determined using UV-visible spectrophotometer (JascoV-730, Tokyo, Japan) at 254 nm.^18^

### Determination of critical micelle concentration (CMC) by conductometry technique

CMC of SOD-CAS was determined as per the reported method.^19,20^ The conductivity was measured using a digital conductometer (Systronics 306, Ahmedabad, India) with conductivity cell (Systronics, Type CD-10, Ahmedabad, India) having platinized electrode with a cell constant of 0.1 to 5.0. All the solutions were prepared in distilled water with different SOD-CAS concentrations. The solutions were stirred for 1 min prior to the conductivity measurement. The conductivity meter was calibrated using standard KCl solution. The accuracy of the conductivity meter was 0.01 µS/cm during the study. The plot SOD-CAS concentration v/s K (conductivity) was checked for linear increase in readings upon addition of SOD-CAS.^19,20^

### Preparation of CLXB loaded CAS carriers

Three methods were used to load CLXB into the carrier CAS, *viz*., nanoparticle preparation, reassembled CAS micelles and CAS nanocapsules.

### Preparation of casein nanoparticles (CNP)

Solutions of SOD-CAS (0.5%, 1% and 1.5% w/v) were prepared in distilled water by stirring. CLXB ethanolic solution (0.8 mL) containing 50 mg of CLXB was added to each 40 mL of the prepared SOD-CAS solutions. The solution was stirred for 1 h to achieve effective CLXB-SOD-CAS binding. Further, 0.5 mL CaCl_2_ solution (1M) was added to this solution. The formed slightly turbid solution was stirred for 30 min. The solutions were centrifuged at 2700 rpm (R-8C, REMI Motors, Mumbai, India) for 10 min to remove the bigger microparticles. Ethanol was removed from the supernatant using a vacuum flash evaporator (ROTAVAP, PBCT-8D, Superfit, India). Supernatant containing nanoparticles was used for further studies. Final CLXB concentration was 1.25 mg/mL.^21^ Due to the addition of CaCl_2_ solution, calcium ions initiate the protein chain rearrangement and the soluble CAS present in the solution turns into the micellar framework and helps in formation of dense nanoparticles.

### Preparation of re-assembled casein micelles (r-CM)

CLXB (50 mg) dissolved in 0.8 mL ethanol was slowly added to 20 mL each of 1%, 2%, 3% w/w SOD-CAS solutions with vigorously stirring. After stirring for 5 min, 0.4 mL of 0.4 M tripotassium citrate, 2.4 mL of 0.08 M dipotassium phosphate (K_2_HPO_4)_ and 2 mL of 0.08 M calcium chloride (CaCl_2_) were added. Eight additions of 0.24 mL 0.08 M K_2_HPO_4_ and 0.50 mL 0.08 M CaCl_2_ were made at 15 min intervals. The pH was maintained between 6.7 and 7.0 with 0.1 N HCl or 1 N NaOH. The CLXB-Caseinate solution was constantly stirred. The final volume was adjusted to 40 mL with distilled water, titrated to a final pH of 6.7 with 0.1 N HCl. The final solutions were further stirred (R-8C, REMI Motors, Mumbai, India) for 1 h to obtain the micellar solutions. Final SOD-CAS concentration was 0.5% (B1), 1% (B2) and 1.5% (B3). The CLXB concentration in each solution was 1.25 mg/mL.^22^ The solvents were eliminated from the suspension using a vacuum flash evaporator (PBCT-8D, ROTAVAP, Superfit, India).

### Preparation of casein nanocapsules (CNC) using soya lecithin

For preparing nanocapsules, the organic phase was formed by dissolving 400 mg of soya lecithin and 50 mg CLXB in 0.8 mL of ethanol, followed by the addition of 0.20 mL of Labrasol and 12.0 mL of acetone. This organic phase was immediately poured over 25 mL each of 0.5% (C1), 1% (C2) and 1.5% (C3) w/v SOD-CAS solutions in 10 mM phosphate buffer (pH 7.4) to obtain the nanocapsule suspension. The solvents were eliminated from the suspension using a vacuum flash evaporator (PBCT-8D, ROTAVAP, Superfit, India). The final volume was 40 mL and CLXB concentration in each solution was 1.25 mg/mL.^23^

### Evaluation of the CAS formulations

#### Entrapment efficiency and drug loading

To 5 mL of each of the prepared formulations, 5 mL SIFSLS 1% was added in a 10 mL test tube. The aqueous suspension was sonicated in a probe ultrasonicator (Oscar Ultra Sonics, Mumbai, India) for 5 min. The formulations containing CLXB were separated from unentrapped CLXB by centrifugation (R-8C, REMI Motors, Mumbai, India) at 9000 rpm for 25 min. The supernatant was recovered and assayed spectrophotometrically using a UV spectrophotometer (V-730, Jasco, Tokyo, Japan) at 254 nm against SIFSLS 1% solution. CLXB concentrations for total and non-encapsulated CLXB samples were estimated. The drug entrapment efficiency was calculated using following equation.^24,25^

$$Entrapment\;efficiency(\%)=\frac{Total\;drug\;added-unentrapped\;drug}{Total\;drug\;added}\times 100$$

The drug loading was determined by centrifugation of 10 mL of formulation at 9000 rpm for 25 min. The unloaded CLXB in the supernatant obtained after centrifugation (R-12C, REMI Motors, Mumbai, India) of all the batches was determined using UV spectrophotometer (V-730, Jasco, Tokyo, Japan) at 254 nm against SIFSLS 1% solution. Percent drug loading for each batch was calculated using following equation.^26^

$$Drug\;loading(\%)=\frac{Total\;drug\;added-unentrapped\;drug(mg)}{Total\;drug\;added(mg)+Total\;excipients\;added(mg)}\times 100$$

### Measurement of particle size and zeta (ζ) - potential 

The zeta (ζ) - potentials and the mean particle size of the CAS aggregates were determined by Zetasizer (v2.3, Malvern Instruments Ltd., Mumbai, India). Measurements were carried out at 25 °C in triplicate.^27^

### In vitro release studies

The formulation (10 mL) was filled into pre-swollen dialysis bags-110 LA 395-1MT (length: 7 cm, molecular weight cut off: 1200-14 000 Dalton (Himedia Laboratories Pvt. Ltd., Mumbai, India) for *in vitro* release studies using USP type-II apparatus (LABINDIA, DS 8000, India). The release studies were carried out at 50 rpm using 400 mL of SIFSLS 1% as dissolution medium.^28,29^ The temperature of the release medium was maintained at 37±0.5ºC. Aliquots (2 mL) were withdrawn at every 10, 20, 30, 40, 50, 60, 90, 120 min and filtered. The same volume was replaced with fresh medium maintained at similar temperature. The same procedure was followed for 10 mL saturated solution of CLXB. The absorbance of filtrate was measured by UV spectrophotometric method (V-730, Jasco, Tokyo, Japan) at 254 nm to estimate CLXB concentration.^30-32^ All the measurements were carried out in triplicate.

### Lyophilization 

Prepared CNP (batch A2 solution) was frozen by freezing in ultra-low freezer (Panasonic, MDF-U55V-PE ULT model, Japan) at -81°C for 24 h and further lyophilized (Labconco, Free Zone 2.5 plus, Missouri, USA). The solutions were freeze dried without any adjuvants. The samples (100 mL) were dispensed in 250 mL semi-stoppered glass beaker and minimum temperature of the lyophilization chamber was maintained at -82°C. Sublimation lasted 48 h at a vacuum pressure of 0.090 mBar without heating, while maintaining condenser surface temperature at -82°C.^33^

### Determination of surface morphology

Solution of CNP (batch A2) and its freeze dried powder were examined under a scanning electron microscope (SEM) (Carl Zeiss AG, Oberkochen, Germany) to study the particle surface morphology and shape. Samples were spread over a slab and dried under vacuum. Micrographs were taken using Supra 5 SEM at an accelerating voltage of 10 kV.^34-36^

### Preparation of physical mixture

CLXB and SOD-CAS were passed through a sieve # 100 and the physical mixture (PM) was prepared by mixing pre-weighed amount of CLXB with SOD-CAS at a 1:1 ratio.^34^

### Fourier-transform infrared study

The Fourier-transform infrared spectra (FTIR) of CLXB, SOD-CAS, PM and freeze dried CNP (batch A2) were recorded over a range of 4000–600 cm^-1^ to study the principal peaks FTIR spectrophotometer (Alpha T, Bruker, Germany) by attenuated total reflection (ATR) method. Processing the FTIR spectrophotometric data was done using OPUS Version - 7.5 software.^37^

### X-ray powder diffraction

The X-ray powder diffraction (XRPD) spectra of CLXB, PM and freeze dried CNP (batch A2) were recorded using a Smart Lab high power powder X-ray diffractometer (Rigaku Corp., Tokyo, Japan) with Cu as a target filter, a voltage/current of 40 kV/40 mA and a scan speed of 4º/min. The samples were analyzed at a 2θ angle range from 10° to 89°. The step time was 0.5 s and the acquisition time was 1 h.^24^

### Differential scanning calorimetry

The samples of CLXB, SOD-CAS, freeze dried CNP (batch A2) and PM were placed in sealed aluminium pans and heated at 10°C/min under a nitrogen atmosphere (flow rate 20 mL/min) in the range of 30-300ºC. Thermograms were recorded using differential scanning calorimeter (4000, Perkin Elmer, Massachusetts, United States). Processing of the calorimetric data was done using PYRIS version - 11.1 software.^38^

### Ex-vivo permeation study

The everted gut sac method was used to determine the apparent permeability of pure CLXB and freeze dried CNP (batch A2).^39,40^ The intestine used was of male White Leghorn chicks weighing between 800 and 1000 g and was bought from a local slaughter house within 30 min of slaughter. By rinsing intestine with a pH 7.4 buffer solution (Krebs–Ringer solution) the lumen was thoroughly removed from the mucus. The ileum bag was cut into equal segments of 6.9 cm length each, flushed with normal saline to remove the contents, and then immersed in SIFSLS 1% solution. Each segment was inverted by gently pushing a notched glass rod through the whole length of the intestine and then filled with 1 mL of Krebs solution.^41^ Both ends of each segment were tied with a thread forming an everted ileum bag. Each bag was immersed in 400 mL SIFSLS 1% solution containing CLXB (12.5 mg) or CLXB loaded CNP equivalent to 12.5 mg at 37°C (outer compartment). Samples were withdrawn at predetermined time intervals for 90 min from inside of the sac. The concentration of CLXB permeated was determined by measuring its absorbance using a UV spectrophotometer at 254 nm and the following equation was used to calculate the apparent permeability.^42-44^

$$Apparent\;Permeability(\frac{mg}{cm^{2}})=\frac{Amount\;of\;CLXB\;permeated(mg)\times Volume}{Surface\;area\;of\;mucous\;membrane}$$

To calculate the surface area (A) of mucus membrane, the intestine was considered a cylinder and the following equation was used:

A(cm^2^) = 2πrh + 2πR^2^

Therefore, the surface area (A) of mucous membrane was 11.61 cm^2^ and the concentration of the CLXB in the donor compartment was 0.125 mg/ml. The linear regression analysis of plot of drug permeated (µg) *vs*. time (min) was used to obtain the slopes for determining flux.

### Comparative in-vitro dissolution studies 

The freeze dried CNP (batch A2) (510 mg) equivalent to 100 mg CLXB was filled in hard gelatin capsule shell, size 0 (Qualicaps Inc., Japan). The dissolution rate of the pure CLXB, filled CNP capsule and Zycel-100 capsule (Zydus Healthcare Limited, India) was determined using USP type-II apparatus (DS 8000, LABINDIA, India) at 50 rpm using 900 mL of SIFSLS 1%. The temperature of the dissolution medium was maintained at 37±0.5°C. Aliquots (5 mL) were withdrawn at 10, 20, 30, 40, 50, 60, 90 and 120 min intervals, filtered and the absorbance of filtered sample solution was measured by UV spectrophotometric method (Jasco, Tokyo, Japan, model V-730) at 254 nm. All the measurements were carried out in triplicate. The concentration of CLXB was determined from the standard calibration curve.

### Analysis of drug release kinetics

To determine the drug release kinetics, the drug release data of CNP (A2 formulation) was analyzed for zero order and first order kinetic equations. Further, to confirm the drug release mechanism, the data was analyzed according to the Higuchi, Hixson-Crowell and Korsmeyer’s-Peppas’ models. Kinetic analyses were performed using PCP-Disso-v3 software.

### Stability testing

Stability studies were carried out according to the ICH Q1A (R_2_) guidelines at different storage temperature and RH conditions (40 ± 2°C and 75 ± 5% RH) and (25 ± 2°C and 60 ± 5% RH) on freeze dried CNP batch A2 using triple stability chamber (Thermolab, Mumbai, India). The product was kept in petri plate. The samples were taken at predetermined time interval (15, 30, 45, 60 and 90 days) to determine the appearance, texture and percentage drug content.^37^ For the estimation of percentage drug content, accurately weighed (10 mg) of CNP formulation was dispersed into 25 ml of SIFSLS 1%. The prepared mixture was shaken for 24 h. After 24 h, the solution was filtered, and the filtrate was analyzed for drug content by a UV spectrophotometer (V-730, Jasco, Tokyo, Japan) at 254 nm after suitable dilution. The drug content was determined using equation: *y = mx ± c*, where *y* is the absorbance, *m* is slope, *x* is drug amount, and *c* is the intercept. Slope and intercept values were taken from the calibration curve.

## Results and Discussion

### Solubility studies

The solubility of CLXB as observed in distilled water, SIFSLS 1% and phosphate buffer (pH 6.8) (Table 1). The results indicated that the highest solubility of CLXB was in SIFSLS 1% and poor solubility was observed in distilled water.

**Table 1:** Results of celecoxib solubility in different solvents and conductometric titration study to determine critical micelle concentration of sodium caseinate

**Solubility of celecoxib**	
**Solvent**	**Solubility (µg/mL)**
Water	6.10 ± 0.033
SIFSLS 1%	240.52 ± 0.098
Phosphate buffer (pH 6.8)	12.01 ± 0.026
**Conductometric titration**	
**Conc. (mg/mL)**	**Conductivity (K) (µS/cm)**
0.01	0.11
0.02	0.28
0.04	0.54
0.06	0.63
0.08	0.69
0.1	0.75
0.12	0.79
0.14	0.83
0.16	0.87
0.18	0.92
0.2	0.97
0.22	1.02

### Critical micelle concentration (CMC)

**Figure 1:**
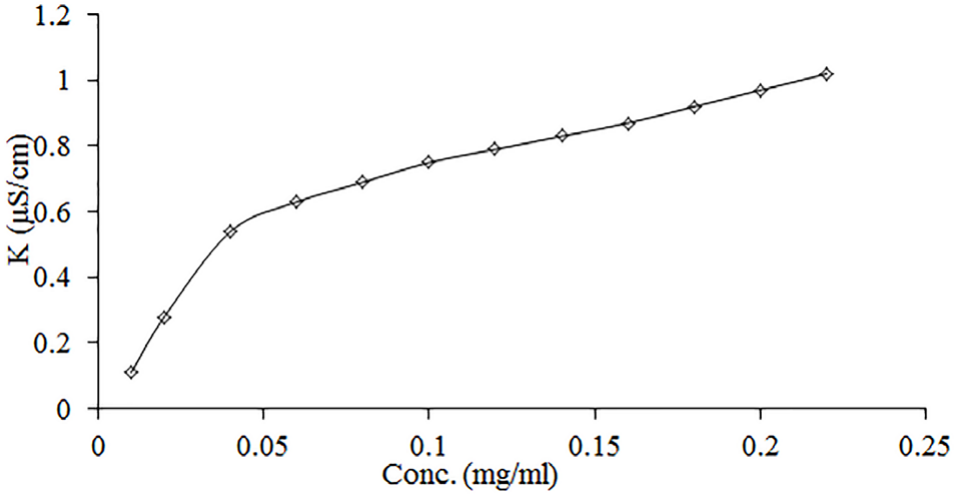
Change of conductivity with increasing SOD-CAS concentration.

Conductivity measurements were used to determine CMC of the negatively charged SOD-CAS. Figure 1 shows the results of conductivity measurements of SOD-CAS solutions. Initially, the conductivity increased linearly upon addition of SOD-CAS due to the increased amount of dissolved anions and cations of SOD-CAS. Above the CMC, SOD-CAS micelles were formed (Table 1). The rate of increase in conductivity was decreased with further addition of SOD-CAS. Thus, a smaller slope value was noticed above the CMC.^20^ Hence, the CMC was found in the range of 0.05-0.22 mg/mL.^42^ All further formulations were prepared using SOD-CAS concentrations above its CMC.

### Evaluation of CAS formulations

#### Entrapment efficiency and drug loading

The percentage entrapment efficiency and drug loading in all nine formulations were calculated (Table 2). It is evident that amongst all formulations, the entrapment efficiency and drug loading were highest in CNP batches as compared to CNC and r-CM. At a concentration of 1% SOD-CAS (batch A2) CNP showed highest entrapment efficiency of 90.71±0.01%.

**Table 2:** Evaluation of casein nanocarriers

**Batch**	**Entrapment Efficiency (%)**	**Drug Loading (%)**	**Particle size (nm)**	**Zeta Potential (mV)**	**Polydispersity index**	**Cumulative release (%) (120 min)**
**Casein nanoparticles (CNP)**						
A1 (0.5%)	72.08±1.2	25.02±0.28	259.06±1	-23.6±0.1	0.436±0.001	78.57±0.7
A2 (1%)	90.71±3.1	14.08±0.16	216.1±2	-24.6±0.1	0.422±0.001	98.17±1.3
A3 (1.5%)	82.26±2.3	10.02±0.14	222.53±4	-24.5±0.1	0.371±0.002	92.84±1.4
**re-assembled casein micelles (r-CM)**						
B1 (0.5%)	72.05±1.2	15.21±0.17	631.8±7	-29.3±0.1	0.786±0.001	76.34±0.6
B2 (1%)	90.65±3.4	9.44±0.12	665.3±6	-28.9±0.5	0.587±0.001	89.24±1.5
B3 (1.5%)	81.14±2.1	6.84±0.11	643.6±8	-29.5±0.1	0.625±0.002	82.40±0.6
**Casein nanocapsules (CNC)**						
C1 (0.5%)	72.03±1.4	8.54±0.12	394.6±5	-32.3±0.1	0.197±0.002	11.85±0.9
C2 (1%)	90.52±3.2	6.94±0.11	389.2±3	-29.5±0.1	0.191±0.001	18.31±1.1
C3 (1.5%)	80.15±2.4	5.85±0.10	396.4±4	-30.5±0.1	0.262±0.001	13.50±0.6

### Particle size and zeta (ζ) - potential

The particle size of different CNP, r-CM and CNC formulations was found to be in the range between 216.3 nm to 665.1 nm (Table 2). The zeta potential of different CNP, r-CM and CNC batches was found to be -23.8 mV to - 32.2 mV (Table 2) indicating a good colloidal stability.^45^ This negative charge is a result of the net electrostatic charge on the CAS surface at pH 7.4, which is above its isoelectric point, where the CAS carboxylic groups become negatively charged.

### In vitro drug release

**Figure 2:**
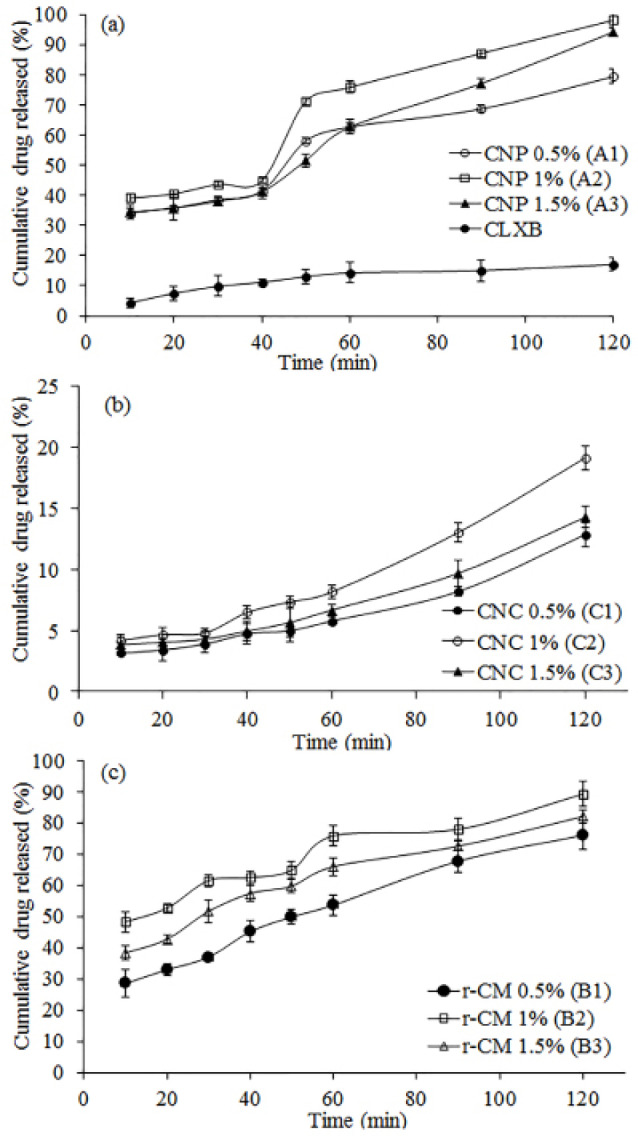
*In vitro* release profile of Celecoxib from CNP (a), CNC (b), and r-CM (c) by dialysis bag method in 400 mL of SIFSLS 1% at 50 rpm and 37±0.5C using USP type-II apparatus. Data presents mean ± SD (n = 3).

*In vitro* drug release studies were carried out in SIFSLS 1%. Results of *in vitro* drug release studies are shown in Table 2. The results show that 17.03% of pure CLXB was released from CNP in 120 min (Figure 2a), suggesting a strong need to enhance the dissolution of CLXB. The presence of SOD-CAS increases the dissolution rate of CLXB up to a drug-excipient ratio of 1:1. Factors such as the lack of crystalline form (supported by XRD results), the increased surface area of the drug, and the hydrophilic surface of SOD-CAS helped to improve the dissolution of CLXB. The highest percentage cumulative release was observed from CNP batches (Table 2 and Figure 2a). Percentage cumulative release for CNC was found to be poor, this might be due to the formation of a viscous boundary layer around the CLXB particles, leading to increase in diffusion path length and decrease in the dissolution rate (Table 2 and Figure 2b). The r-CM formulations showed higher percentage cumulative drug release as compared to CNC batches. The highest percentage cumulative release was 89% at 1% concentration of SOD-CAS. From the results obtained, it is evident that amongst all formulations the onset of dissolution of CNP batches was higher as compared to CNC and r-CM (Figure 2c). At a concentration of 1% SOD-CAS (CNP batch A2), highest release of 98% was achieved.

### Lyophilization

CAS nanoparticles are not stable in aqueous form, therefore; we examined the ability to form a freeze-dried powder having a much better stability profile. No additives are needed for efficient lyophilization as it has been reported that CAS itself acts as a cryoprotectant.^33^ By lyophilization, without any additives, we are able to keep the CNP nanoparticles (batch A2) stable in a dry powder form for at least 3 months. After freeze-drying of the nanoparticles and re-suspending dried powder in SIFSLS 1% of the original concentration, transparent solutions were formed, that resemble very much the original suspension.^34^

### Surface morphology

**Figure 3:**
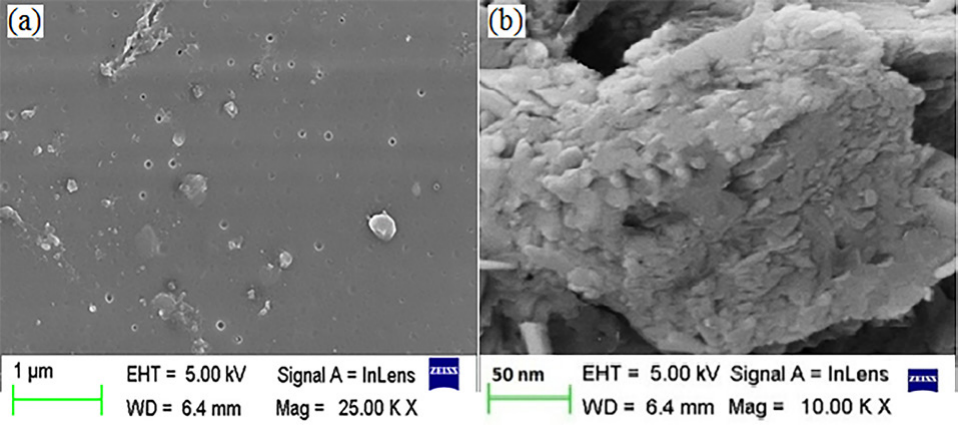
Scanning electron micrograph of CNP suspension (batch A2) (a) and freeze dried CNP (batch A2) (b). Scales are given on individual micrograph.

The SEM of the suspension (CNP batch A2) helps in concluding that the formed nanoparticles are globular, uniform in size and well separated (Figure 3a). In the micrograph of freeze dried powder CNP, the original morphology of the raw materials disappeared, and it was not possible to differentiate the two components (Figure 3b). The freeze dried samples appeared as agglomerates. The amorphous form of the freeze dried sample was indicative of the presence of a new solid phase, leading to estimate the existence of a single phase.^46^

### Fourier-transform infrared study

**Figure 4:**
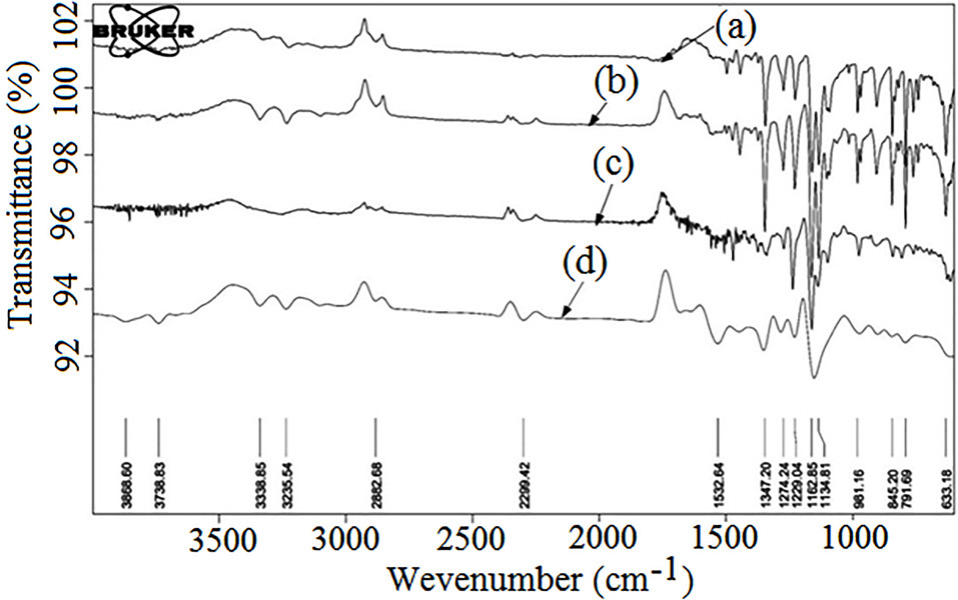
Fourier transform-infrared spectra of CLXB (a), physical mixture of CLXB and SOD-CAS (b), SOD-CAS (c), and freeze dried CNP batch A2 (d).

The physical mixture was prepared to check for possible interactions between CLXB and SOD-CAS. In the FTIR spectrum of CLXB, the medium absorption bands at 3160 cm^-1^ and 3260 cm^-1^ are assigned to -NH symmetric and asymmetric stretching vibrations, respectively (Figure 4a). The other characteristic bands are attributed to: 1150 and 1343 cm^-1^ (S=O symmetric and asymmetric stretching, respectively), 1532 cm^-1^ (NH bend), 3222 cm^-1^ (NH_2_ stretching) and 838 cm^–1^ (CH bend).^47^ The FTIR spectrum of PM showed compatibility of drug CLXB with CAS (Figure 4b). The spectrum of SOD-CAS showed important bands at 1159 cm^-1^ (C-O stretch), 903-074 cm^-1^ (C-H bend) and 1844 cm^-1^ (C=O) (Figure 4c). The softening of peaks in the spectrum of freeze dried CNP indicated the encapsulation of CLXB in CNP (Figure 4d).

### X-ray powder diffraction (XRPD)

XRPD studies were carried out for further characterization of solid states of the samples. The patterns obtained for pure CLXB, PM and lyophilized powder of CNP (batch A2) are depicted inFigure 5 (top panel). The characteristic diffraction peaks were observed at 2θ values of 10.74°, 14.85°, 16.14°, 22.16°, 27.02°, and 30.38° correspond to the powder diffraction pattern for pure CLXB. The crystalline state of CLXB in the physical mixture of SOD-CAS and CLXB is evident from the characteristic diffraction peaks. However, the XRPD pattern in the lyophilized powder of CNP suggests the amorphous structure of CLXB within the formulation. These XRPD results are compatible with the DSC observations.

**Figure 5:**
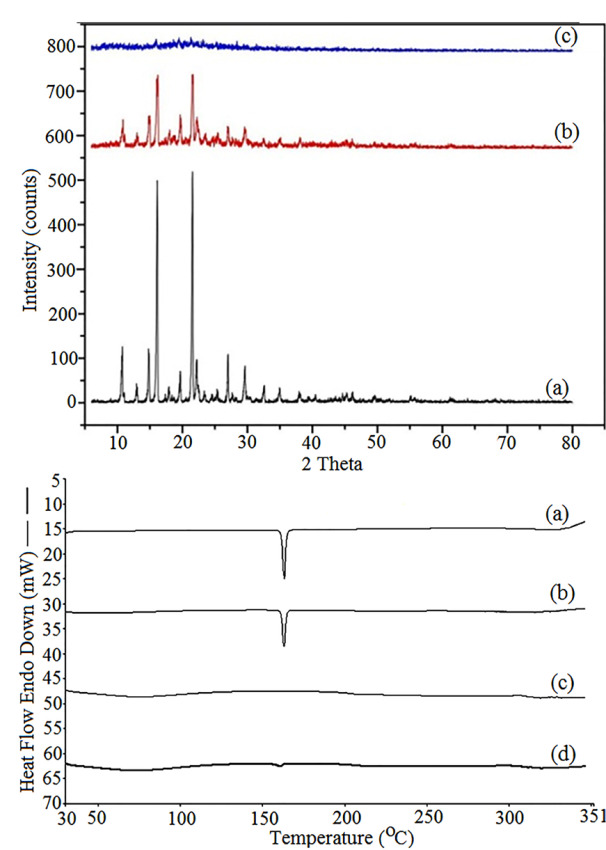
XRD spectrum (top panel): pure Celecoxib (a), physical mixture of CLXB and SOD-CAS (b), freeze dried powder CNP (c), and DSC thermograms (bottom panel): pure CLXB (a), physical mixture of CLXB and SOD-CAS (b), SOD-CAS (c), and CNP (d).

### Differential scanning calorimetry

DSC thermograms and data of pure CLXB, PM, SOD-CAS, and CNP (batch A2) are shown in Figure 5 (bottom panel). Thermogram of CLXB exhibited a sharp endothermic peak at 163.42°C, (ΔH = 131.2 J/g), Tm corresponding to its melting point,^48,49^ indicating crystalline nature of CLXB. Thermogram of PM showed the endothermic peak of CLXB but with a slight shift to lower Tm (163.02°C, at ΔH = 81.06 J/g) that does not seem to be significant indicating compatibility between CLXB and SOD-CAS. Thermogram of SOD-CAS showed no peak, indicating the amorphous nature of SOD-CAS. The lyophilized powder showed a smooth thermal curve and indicating amorphous nature of CLXB. The DSC results confirm the encapsulation of CLXB in nanoparticles. The amorphous property of CLXB in the prepared nanoparticles containing SOD-CAS is responsible for the dissolution enhancement.^48^

### Ex-vivo permeation study

Oral bioavailability is maximum for the drugs having optimum permeability and solubility.^38^ It is indispensable to examine permeation through the mucous membrane to know not only the diffused amount of CLXB, but also to verify whether the release mechanism is influenced by the solubility. It has been reported that the chicken small intestine could be a useful model in permeation studies.^40^ In the present study, the permeation studies were carried out for pure CLXB and optimized CNP formulation (batch A2).

**Figure 6:**
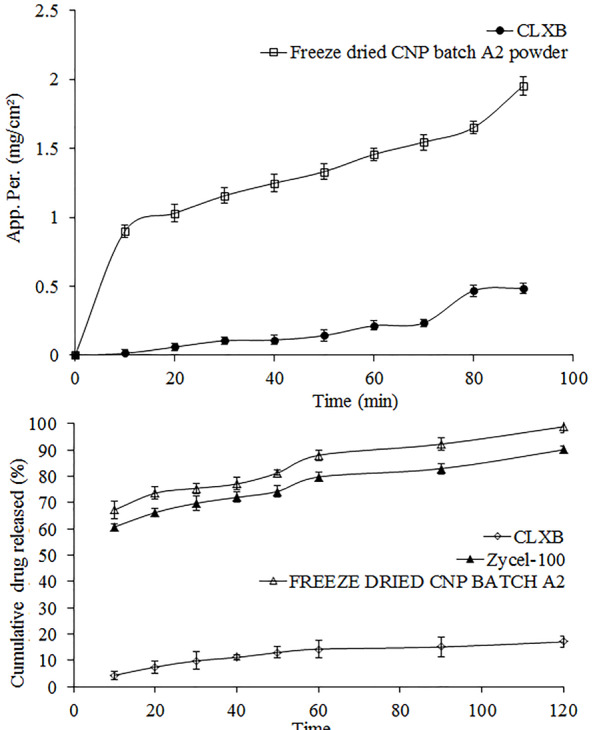
Top panel: comparative apparent permeability of Celecoxib from pure Celecoxib and freeze dried CNP (batch A2) by everted gut sac method in in SIFSLS 1% solution. Bottom panel: **c**omparative*in vitro* release profile of CLXB from pure CLXB, marketed formulation and freeze dried CNP (batch A2) by dialysis bag method in 900 mL of SIFSLS 1% at 50 rpm and 37 ± 0.5 °C using USP type-II apparatus. Data presents mean ± SD (n = 3).

The apparent permeability for both the pure CLXB and the freeze dried CNP powder followed a similar pattern over time (Figure 6). The apparent permeability of the pure CLXB gradually increased from 0.014 mg/cm^2^ after a period of 10 min to a maximum of 0.48 mg/cm^2^ over the subsequent 90 min. Compared to the pure CLXB, the apparent permeability of freeze dried CNP was higher and gradually increased from 0.90 mg/cm^2^ after 10 min to a maximum of 1.95 mg/cm^2^ over the subsequent 90 min. The gradient of the graph of freeze dried CNP was higher than that of the pure CLXB. A higher permeation was recorded at each time point than that of the pure CLXB (Table 3). Nanoparticles solubilize CLXB in the outer compartment, thereby maintaining/increasing the driving force for permeation. This indicated the permeation enhancing potential of molecularly dissolved and solubilized CLXB in the SOD-CAS nanoparticles.

**Table 3:** Results of permeation study of Celecoxib through chicken ileum after 90 min

**Parameters**	**Sample code**	
	**Celecoxib**	**Formulation CNP (batch A2)**
Cumulative amount of Celecoxib permeated (%)	14.7	63.85
J (µg/cm^2^/h)	1.81	3.67

### Comparative in-vitro dissolution studies

Comparative*in vitro* dissolution profiles of pure CLXB, freeze dried CNP (batch A2) filled in hard gelatin capsule and reference formulation (Zycel-100) are presented in Figure 6. The cumulative percentage drug release from the freeze dried CNP and Zycel-100 capsule was 98.83 and 90.05%, respectively after 120 min. The pure CLXB showed only 17.03% of release after a period of 120 min. It is evident that capsule containing freeze dried CNP had improved dissolution rate of CLXB.

### Drug release kinetics

The highest correlation coefficient serves as an indicator of the best fit for each of the models considered. The value of n is about 0.45 for a Fickian release, and for an anomalous or non-Fickian release the release is mainly by diffusion with n values > 0.45 and < 1.0. In the present study, the highest correlation coefficient (R^2^) for Korsmeyer’s-Peppas’ model was 0.9989 and this was the best fit model. The value of release exponent (n) and release rate constant (k) for best fit model were 0.6865 and 0.1770, respectively. It indicated coupling of diffusion and erosion mechanism - so-called anomalous diffusion. This indicated that the drug release was controlled by more than one process. Thus, the *in vitro* release data of optimized batch CNP (batch A2) followed non Fickian diffusion mechanism. This kind of release behavior could be explained due to the erosion or relaxation of casein nanoparticle matrix that would favor drug diffusion. A similar explanation has been proposed for the release of resveratrol from CNP.^50^ Similar release behavior of resveratrol from zein based-nanoparticles has been reported earlier.^51^

### Stability testing 

The lyophilized freeze dried CNP formulation (batch A2) was examined in order to get an idea of any possibility of drug degradation during storage. The results of stability study of various formulations under different conditions are presented in Table 4. As time increased, the drug content decreased in 3 months to 90% under both the study conditions. However, no significant difference in the physical appearance was observed after a period of 3 months.

**Table 4:** Results of stability testing of freeze dried CNP (batch A2) analysis after 90 days study

**Parameters**	**40 ± 2ºC & 75 ± 5% RH**		**25 ± 2ºC & 60 ± 5% RH**	
	**Initial**	**After 90 days**	**Initial**	**After 90 days**
Drug content (%)	100 ± 0.01	90.56 ± 0.01	100 ± 0.01	90.40 ± 0.01
Appearance	Transparent solution	Transparent solution	Transparent solution	Transparent solution

## Conclusion

The SOD-CAS based nanoparticles had spherical shape and uniform size. CLXB-loaded CNP enhanced the dissolution rate of CLXB as compared to both reference product and pure CLXB. The CAS seems to be an appropriate and effective carrier for solubility enhancement of CLXB. The study concludes that the nanometric particles can be freeze-dried without any cryoprotectant and when re-suspended their structural characteristics are well preserved.

## Ethics Issues

Not Applicable.

## Conflict of Interest

The authors confirm that this article has no conflict of interest.

## Acknowledgments

The authors are grateful to Aarti Drugs Ltd., Tarapur, India and Clarion Casein Ltd., North-Gujarat, India for providing CLXB and SOD-CAS samples, respectively to carry out this study.
